# Human endothelial cells secrete neurotropic factors to direct axonal growth of peripheral nerves

**DOI:** 10.1038/s41598-017-04460-8

**Published:** 2017-06-22

**Authors:** Jonathan M. Grasman, David L. Kaplan

**Affiliations:** Department of Biomedical Engineering, Tufts University, Medford, MA 02155 USA

## Abstract

Understanding how nerves spontaneously innervate tissues or regenerate small injuries is critical to enhance material-based interventions to regenerate large scale, traumatic injuries. During embryogenesis, neural and vascular tissues form interconnected, complex networks as a result of signaling between these tissue types. Here, we report that human endothelial cells (HUVECs) secrete brain-derived neurotrophic factor (BDNF), which significantly stimulated axonal growth from chicken or rat dorsal root ganglia (DRGs). HUVEC-conditioned medium was sufficient to enhance axonal growth, demonstrating that direct cell-cell contact was not required. When BDNF was neutralized, there was a significant reduction in axonal growth when incubated in HUVEC-conditioned medium and in direct co-culture with HUVECs. These data show that HUVECs secrete neurotrophic factors that significantly enhance axonal growth, and can inform future *in vivo* studies to direct or pattern the angiogenic response in regenerating tissues to encourage re-innervation.

## Introduction

There are approximately 300,000 cases of peripheral nerve injury each year in Europe and 200,000 cases per year in the United States as a result of traumatic injury, cancer ablation, or cosmetic procedures^1^. Many of these injuries are characterized by volumetric muscle loss (VML), where large amounts of skeletal muscle, blood vessels, nerves, and its native basement membrane are removed or destroyed^2, 3^. Nerve injuries or resections several millimeters in length can regenerate spontaneously^4, 5^; however, traumatic injuries such as those presenting from VML cannot spontaneously regenerate and result in the loss of motor, sensory, and autonomic functions distal to the injured nerve. The current standard of care for peripheral nerve injury is an autologous nerve transplantation, typically from the sural, saphenous, or medial cutaneous nerves^1^. While studies utilizing autologous nerve transplantations have reported complete restoration of sensation, less than 40% of motor function is recovered^6^. Additional complications involved with autografts include loss of function (sensation and/or motor) at the donor site, limited availability of donor nerve tissue, and donor site morbidity^5^. Understanding how nerves spontaneously innervate tissues or regenerate small injuries is critical to developing successful strategies and approaches to enhance current material-based interventions to regenerate large scale, traumatic injuries.

Tissue engineered peripheral nerve regeneration strategies consist of hollow tubes constructed from a variety of polymers including polycaprolactone and silk^5, 7^. The bioactivity of these devices are often enhanced by the incorporation of different extracellular matrix components or growth factors^5^. While these scaffolds have shown partial success *in vivo*, they are limited by the ability of peripheral nerves to regenerate large injuries (>1 cm)^1, 8^. Native nerve regeneration occurs through chemotactic signaling from Schwann cells, facilitating axonal regeneration along the path of the nerve before the traumatic injury. However, once the Schwann cells are damaged, or the injury is too large, axons are unable to bridge these defects, and often will die or innervate incorrect tissues^9^.

An alternate approach to determine strategies for axonal regeneration is to observe events in embryonic development, where neural and vascular tissues form interconnected, complex networks in a coordinated manner^10^. Rather than by two discrete signaling mechanisms, there is significant crosstalk between these pathways both in development and regeneration^11, 12^. For example, in addition to stimulating angiogenesis, vascular endothelial growth factor (VEGF) has been shown to promote neuron survival and is a chemotactic agent to stimulate axonal growth^13, 14^. Endothelial cells secrete factors such as glial-derived neurotrophic factor (GDNF) to enhance neuron survival and axonal growth^15^. Neurotrophic soluble factors such as nerve growth factor (NGF) also stimulate angiogenesis, demonstrating significant overlap between these signaling pathways. The dynamic relationship between vascular and neural tissues persists in adult tissues, where blood brain barrier models show active signaling between these cell types via soluble factors^16^. In fact, there is evidence that neural stem cells stimulate tube formation in populations of endothelial cells^17^, suggesting that this crosstalk works both ways: neural populations stimulate endothelial populations and *vice versa* in adult tissues.

In this study, we investigated the ability of endothelial cells to stimulate axonal growth of dorsal root ganglion (DRG) explants isolated from both chicks and rats to determine how vascular systems stimulate the growth of peripheral nerves. Endothelial cells co-cultured with explants significantly enhanced axonal growth with respect to growth on poly-D-lysine (PDL) coated well plates. Direct cell-cell contact was not required for enhanced axonal outgrowth, endothelial cell conditioned medium was sufficient to enhance growth on PDL coated well plates. We hypothesized that accelerated DRG axonal growth was mediated via the secretion of neurotrophic factors, and identified several growth factors that may contribute to endothelial cell mediated axonal growth. Specifically, neutralization of brain-derived growth factor (BDNF) was found to eliminate the positive effects of endothelial cell conditioned medium, supporting this hypothesis. This facile co-culture system could be used to study the interactions between endothelial and neural cells in a high throughput manner. Additionally, these data should allow for the development of complex *in vitro* model systems to facilitate the study of angiogenesis and neurogenesis and will inform future tissue engineering strategies to direct targeted innervation of regenerating or transplanted tissues.

## Results

### Surface Composition Affects Axonal Growth of Dorsal Root Ganglia (DRGs)

**Figure 1:**
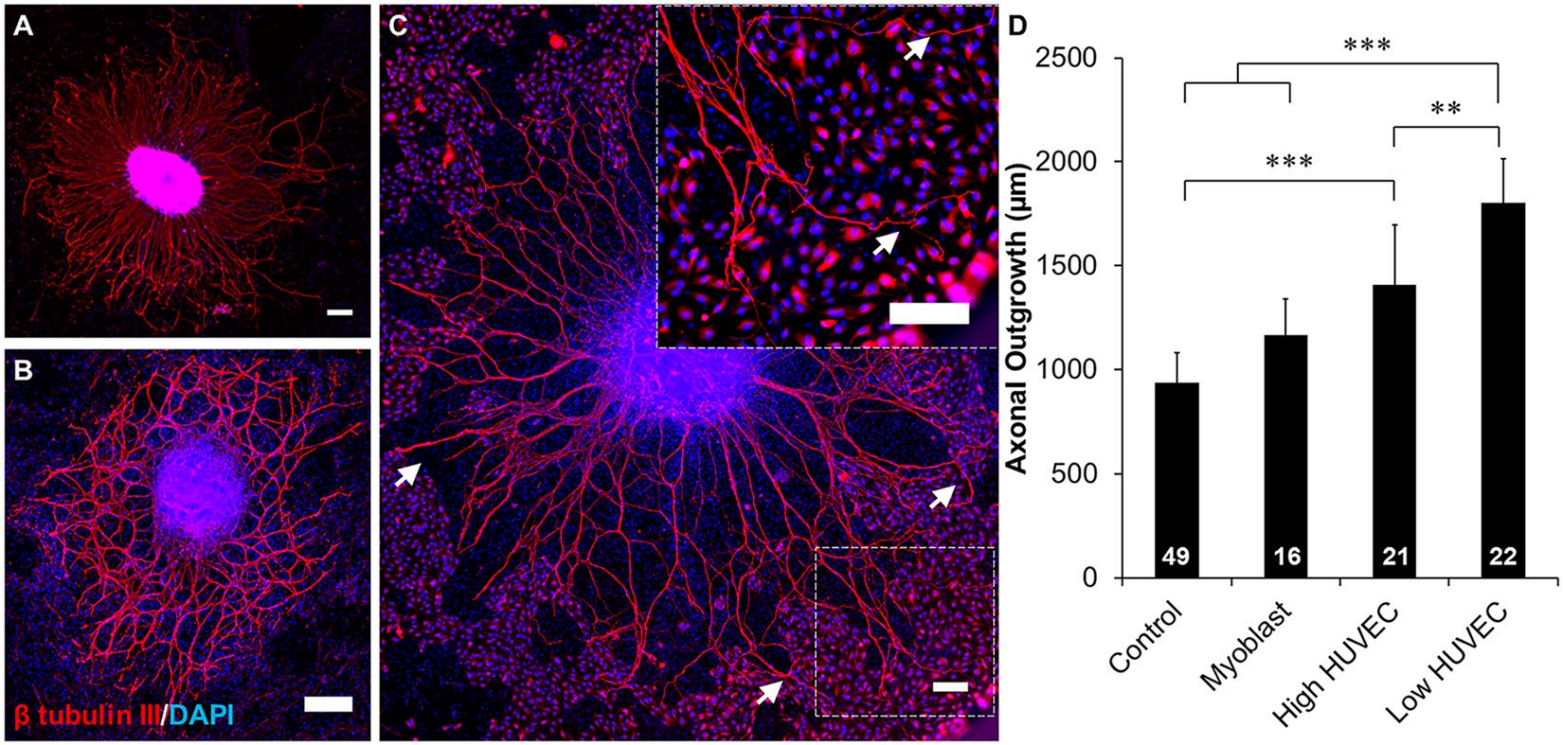
HUVEC enhanced DRG axonal growth is cell type specific. DRG explants cultured on (**A**) poly-D-lysine (PDL) coated wells, (**B**) myoblasts, and (**C**) HUVECs. PDL and myoblast coatings facilitated axonal growth that was radially symmetrical while HUVEC co-cultures facilitated asymmetric growth towards clusters of HUVECs (arrows). Scale = 200 µm. (**D**) Quantification of axonal growth from DRG explants on different surfaces. Low density of HUVECs supported the most axonal outgrowth of all treatment groups. Data are presented as mean ± standard error. *(p < 0.05), **(p < 0.01), ***(p < 0.001) and brackets indicate significance from other surfaces as determined by one-way ANOVA with Holm-Sidak post hoc analysis (DRG sample size for each group shown in bars from 3 independent replicates).

The growth of dorsal root ganglia (DRGs) on different surfaces, either matrix-based or cell-based, was investigated to evaluate the ability of different cell types to enhance axonal growth. Axons grew out of the DRG body with radial symmetry on poly-D-lysine (PDL) coated wells and myoblasts (Fig. 1A,B). In contrast, there appeared to be some degree of asymmetry of axonal growth on HUVEC co-cultures, with axons growing towards clusters of HUVECs (Fig. 1C). Asymmetric and HUVEC cell-guided axonal growth was more pronounced when the density of HUVECs was decreased tenfold (low density HUVEC). Axon length was found to be significantly longer in both HUVEC co-culture conditions than in monoculture on PDL (Fig. 1D). Axonal growth was significantly longer when co-cultured with low densities of HUVECs with respect to co-cultures of high densities of HUVECs or myoblasts and monocultures on PDL. DRGs were also grown on C2C12 myoblasts to confirm the specificity of HUVEC-mediated axonal growth. Axons were observed to be in close proximity to developing myotubes, and exhibited a branching morphology indicative of neuromuscular junction (NMJ) formation. These NMJ structures stained positive for α-bungarotoxin, which binds to acetylcholine receptors, further supporting the hypothesis that these structures are NMJs and that they can form after as little as 4 days of co-culture (Supplemental Figure S1). Despite the coupling identified between these two cell types, there was no significant increase in axonal growth when co-cultured with myoblasts.

### HUVEC Secrete Cytokines that Enhance Axonal Growth

**Figure 2:**
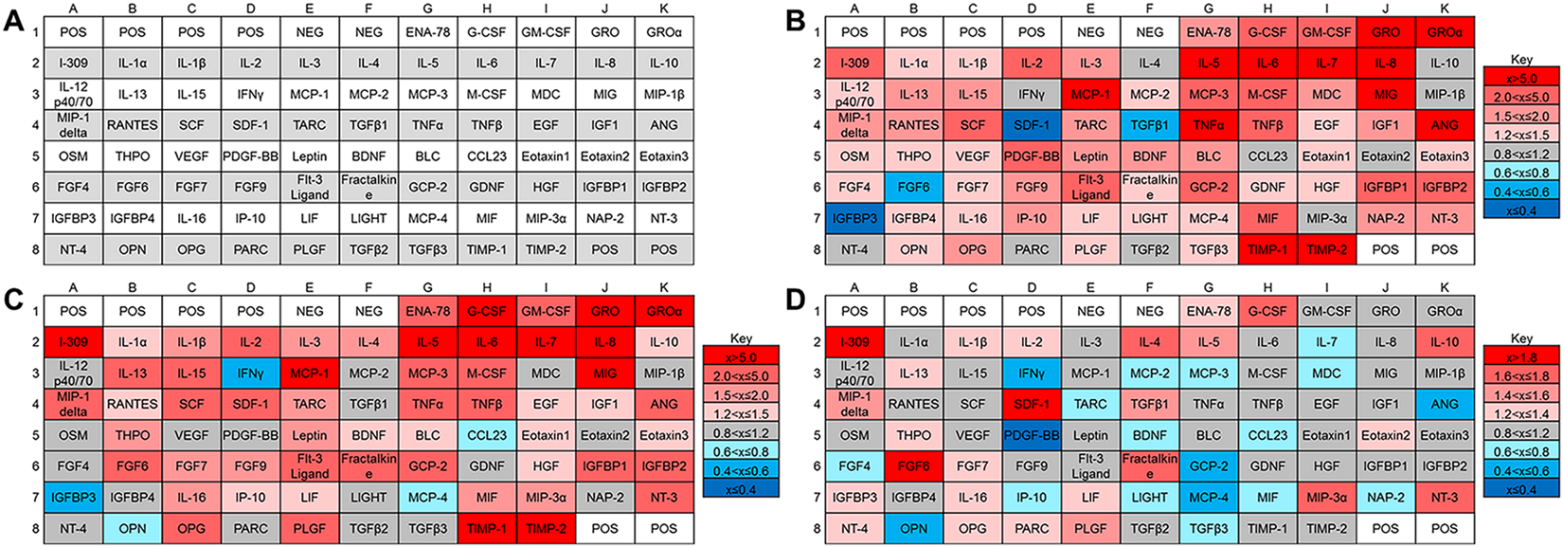
HUVECs and DRGs secreted a variety of proteins. (**A**) Map of cytokine array used to determine protein secretion from (**B**) HUVEC monocultures and (**C**) DRG-HUVEC co-cultures normalized to EGM-2 medium. To determine how the DRG population affected the secretion and consumption of proteins, (**D**) results from arrays assayed with DRG-HUVEC co-cultures were normalized to HUVEC monocultures. In all cases, red indicates overexpression of indicated proteins and blue indicates underexpression of indicated proteins, with gradations of each color indicating relative amount of over/under expression. POS and NEG wells are internal controls within the cytokine array assay. Data are compiled from two independent replicates.

To determine what cytokines and growth factors were secreted from HUVECs, a commercially available cytokine array was used to detect the secretion profile of 80 different factors, including neurotrophic and angiogenic growth factors, in HUVEC conditioned medium (Fig. 2A). A majority of the cytokines was overexpressed in HUVEC conditioned medium (Fig. 2B) and HUVEC-DRG conditioned medium (Fig. 2C) when normalized with blank EGM-2 medium. When the HUVEC-DRG co-culture conditioned medium was normalized against the HUVEC conditioned medium (Fig. 2D), there were 10 unique cytokines that were overexpressed: stromal derived factor 1 (SDF1), fibroblast growth factor 6 (FGF6), transforming growth factor β1 (TGFβ1), insulin-like growth factor binding protein 3 (IGFBP3), macrophage inflammatory protein 3α (MIP3α), interleukin 10 (IL10), IL4, neurotrophin-3 (NT3), eotaxin2, and pulmonary and activation-regulated cytokine (PARC). Nineteen cytokines were underexpressed, indicating a consumption of these factors by the DRG cell population: brain derived neurotrophic factor (BDNF), FGF4, IL7, migration inhibitory factor (MIF), osteopontin (OPN), platelet-derived growth factor ββ (PDGF-ββ), monocyte chemoattractant protein 3 (MCP3), interferon gamma-induced protein 10 (IP10), MCP2, macrophage-derived cytokine (MDC), MCP4, TGFβ3, angiotensin (ANG), thymus and activation-regulated chemokine (TARC), LIGHT, neutrophil activating peptide 2 (NAP2), granulocyte chemotactic protein 2 (GCP2), CCL23, and interferon gamma (IFNγ).

**Figure 3:**
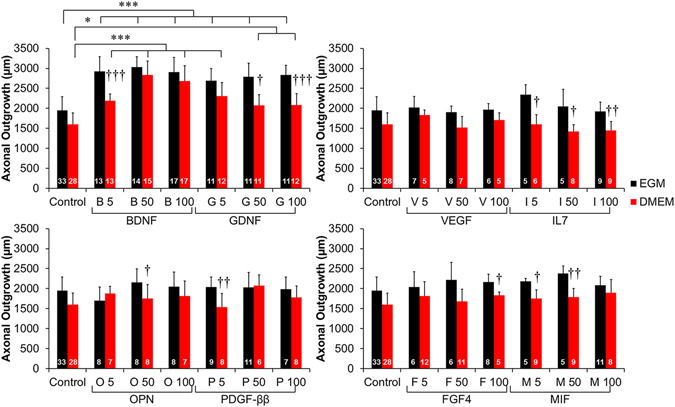
Neurotrophic factors secreted by HUVECs enhance axonal growth from DRG explants. Seven factors that were secreted by HUVECs and underexpressed in HUVEC-DRG co-cultures were selected from the results of the cytokine array. DRGs were cultured on PDL coated wells in either EGM-2 or DMEM medium supplemented with 0, 5, 50, or 100 µg/mL of BDNF, GDNF, VEGF, IL7, OPN, PDGF-ββ, FGF4, or MIF for 4 days. All concentrations of BDNF and GDNF significantly enhanced axonal outgrowth. Axonal outgrowth was not affected by the concentration of the remaining growth factors, although there were some treatment groups, such as IL7, that displayed significantly higher axonal outgrowth when cultured in EGM-2 rather than DMEM. Data are presented as mean ± standard error. ^†^(p < 0.05), ^††^(p < 0.01), ^†††^(p < 0.001) indicates significance from EGM-2 medium by two-tailed Student’s t-test; *(p < 0.05), **(p < 0.01), ***(p < 0.001) and brackets indicate significance from indicated treatments as determined by one-way ANOVA with Holm-Sidak post hoc analysis (DRG sample size for each group shown in bars from 2 independent replicates). Comparisons of all other growth factors to controls were not significant (ANOVA).

To identify which of these factors might be playing a role in axonal growth, we focused on cytokines that were underexpressed in the array, under the assumption that underexpression was a result of the factors being consumed by the cells within the DRG explants. From the 19 underexpressed cytokines, we selected 7 based on a literature search: BDNF, FGF4, IL7, VEGF, MIF, OPN, and PDGF-ββ. DRGs were cultured on PDL coated wells in the presence of 0, 5, 50, or 100 µg/mL of each growth factor, and the axonal growth was determined. Glial derived neurotrophic factor (GDNF) was used as a positive control, as it was found to be secreted by HUVECs and has been used to enhance peripheral nerve regeneration *in vivo* ^18–20^. This experiment was performed complete DMEM as well as HUVEC culture medium (EGM-2) to determine if the culture medium affected axonal growth (Fig. 3). There were no significant differences in control conditions between the two different cell media.

All concentrations of BDNF significantly lengthened the axonal outgrowth of DRGs, and interestingly axonal outgrowth was significantly increased in EGM-2 medium compared to DMEM with 5 µg/mL of BDNF as well as the higher concentrations of GDNF (50 and 100 µg/mL). While there were qualitative increases in axonal length with supplementation of 5 µg/mL of IL7, 50 µg/mL of MIF, and 50 µg/mL of FGF4, none of these treatment groups significantly increased axonal length. Supplementation of DRG cultures with VEGF, OPN, and PDGF-ββ did not show any concentration-dependent increase in axonal growth, despite axonal growth being qualitatively longer when grown in EGM-2 rather than DMEM.

**Figure 4:**
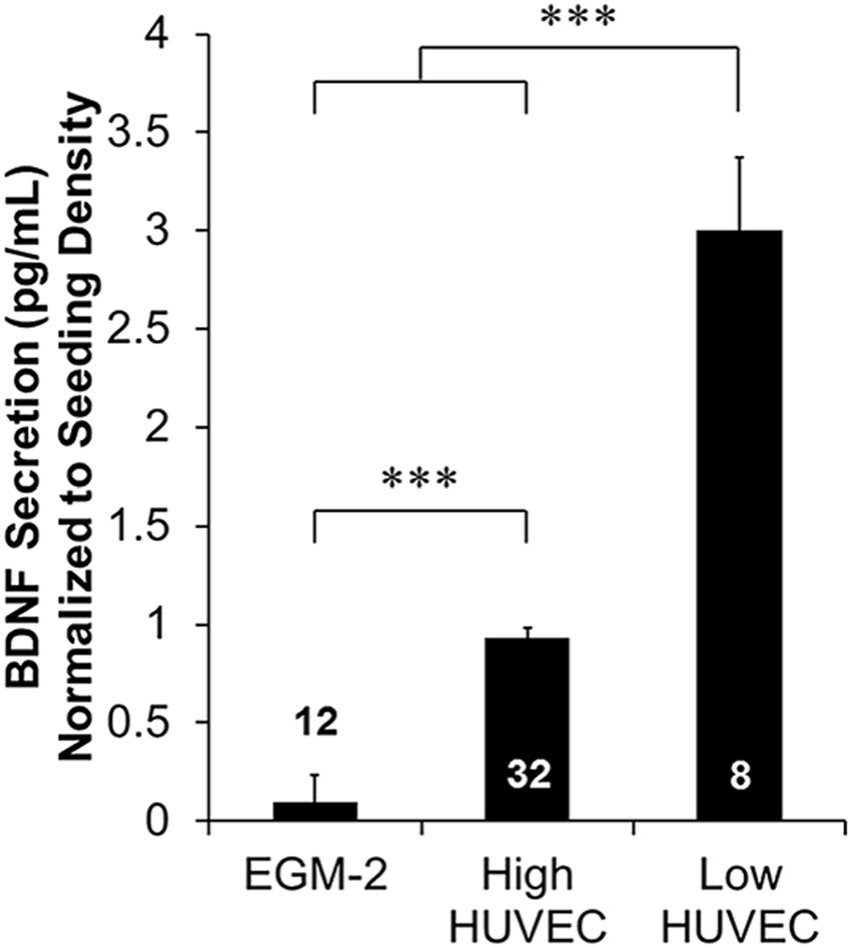
Quantification of HUVEC secretion of BDNF. HUVECs were seeded at high (10,000 cell/cm^2^) or low (1,000 cell/cm^2^) densities and cultured in parallel with DRG-HUVEC co-cultures and assayed for BDNF using ELISA kits, and normalized to their respective seeding densities. Both concentrations of HUVECs secreted measurable amounts of BDNF significantly higher than EGM-2 and low density seeding of HUVECs produced higher concentrations of BDNF per cell than high density seeding of HUVECs. Data are presented as mean ± standard error. ***(p < 0.001) and brackets indicate significance from other treatments as determined by one-way ANOVA with Holm-Sidak post hoc analysis (Well sample size for each group shown in bars from 2 independent replicates).

To quantify the amount of BDNF that was secreted by HUVECs, an ELISA was performed on HUVECs grown in monoculture. High density HUVEC cultures secreted 37.36 ± 1.94 pg/mL BDNF, which was significantly higher than the amount of BDNF secreted from low density HUVEC cultures (12.00 ± 1.50 pg/mL). After normalization to HUVEC seeding density, low density HUVECs secreted significantly higher amounts of BDNF per cell (3.00 ± 0.37 pg/mL) than high density HUVECs (0.93 ± 0.05 pg/mL) (Fig. 4).

### Neutralization of Neurotrophic Factors Limits HUVEC-Mediated Axonal Growth

**Figure 5:**
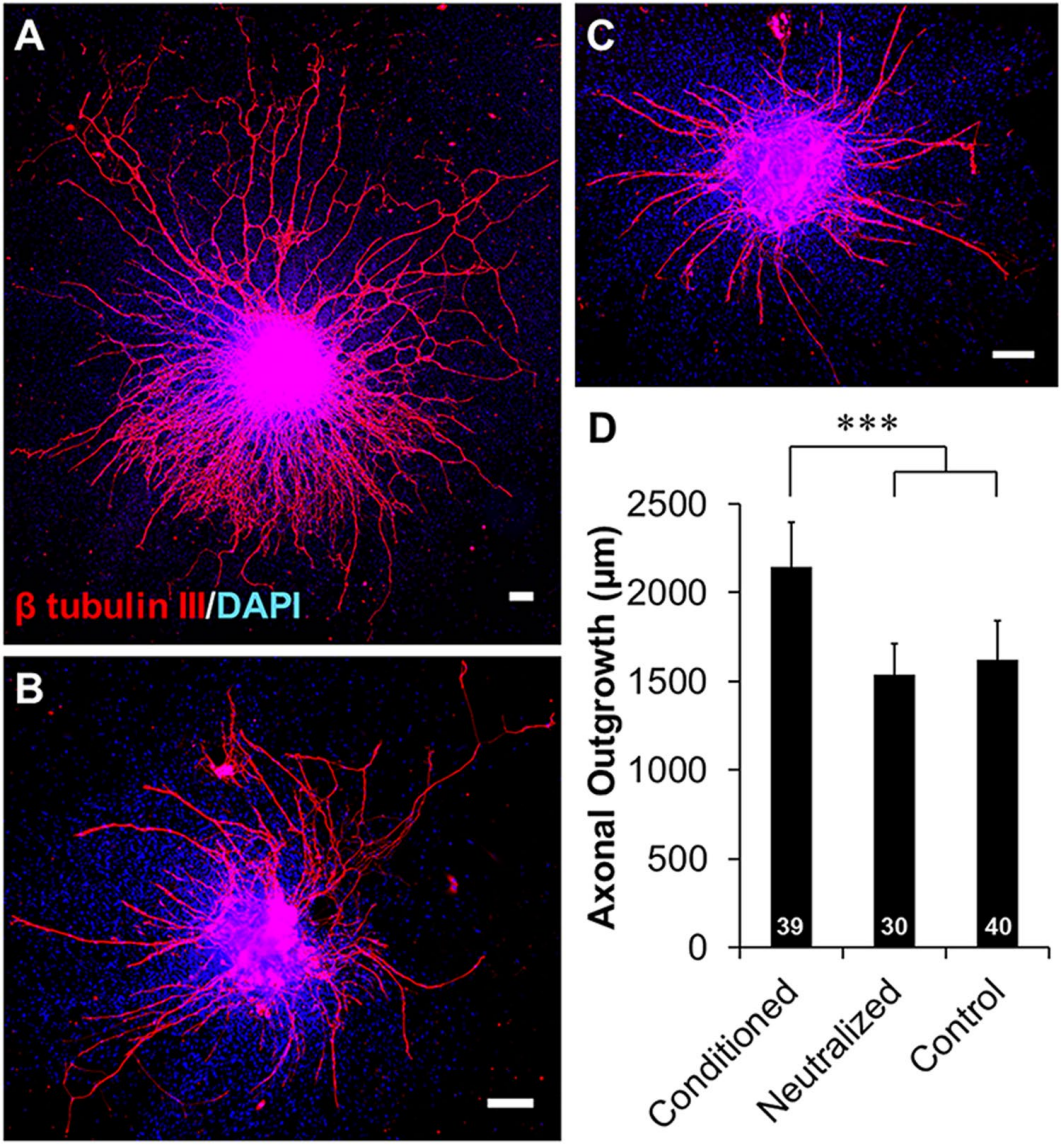
Neutralization of BDNF reduced DRG axonal growth in HUVEC-conditioned medium. DRG explants were cultured on PDL coated wells in (**A**) HUVEC-conditioned medium, (**B**) HUVEC-conditioned medium supplemented with 125 ng/mL of BDNF neutralizing antibody, and (**C**) non-conditioned EGM-2 medium (control). HUVEC-conditioned medium increased the amount of axonal branching from DRG explants. Scale = 200 µm. (**D**) Quantification of axonal growth between HUVEC-conditioned medium, neutralized conditioned medium, and control. Neutralization of BDNF negated the significant increase of axonal growth observed from conditioned medium. Data are presented as mean ± standard error. ***(p < 0.001) and brackets indicate significance from other treatments as determined by one-way ANOVA with Holm-Sidak post hoc analysis (DRG sample size for each group shown in bars from 3 independent replicates).

To confirm that BDNF was necessary for HUVEC-mediated axonal growth, DRGs were grown in HUVEC-conditioned medium or in direct co-culture with HUVECs in the presence of BDNF neutralizing antibody, which has been previously validated to efficiently knockdown BDNF activity^21–23^. HUVEC-conditioned medium supported robust axonal growth from DRGs (Fig. 5A) and increased the amount of axonal branching, with respect to control DRGs (Fig. 5C). The addition of BDNF neutralizing antibodies significantly reduced axonal growth and branching from the control DRGs (Fig. 5B). DRGs cultured in HUVEC-conditioned medium supported significantly more axonal outgrowth than control DRGs or DRGs grown in neutralized conditions (Fig. 5D). Supplementation with neutralizing antibodies completely negated the enhancing effects of HUVEC-conditioned medium, bringing values of axonal growth back to levels observed from controls.

**Figure 6:**
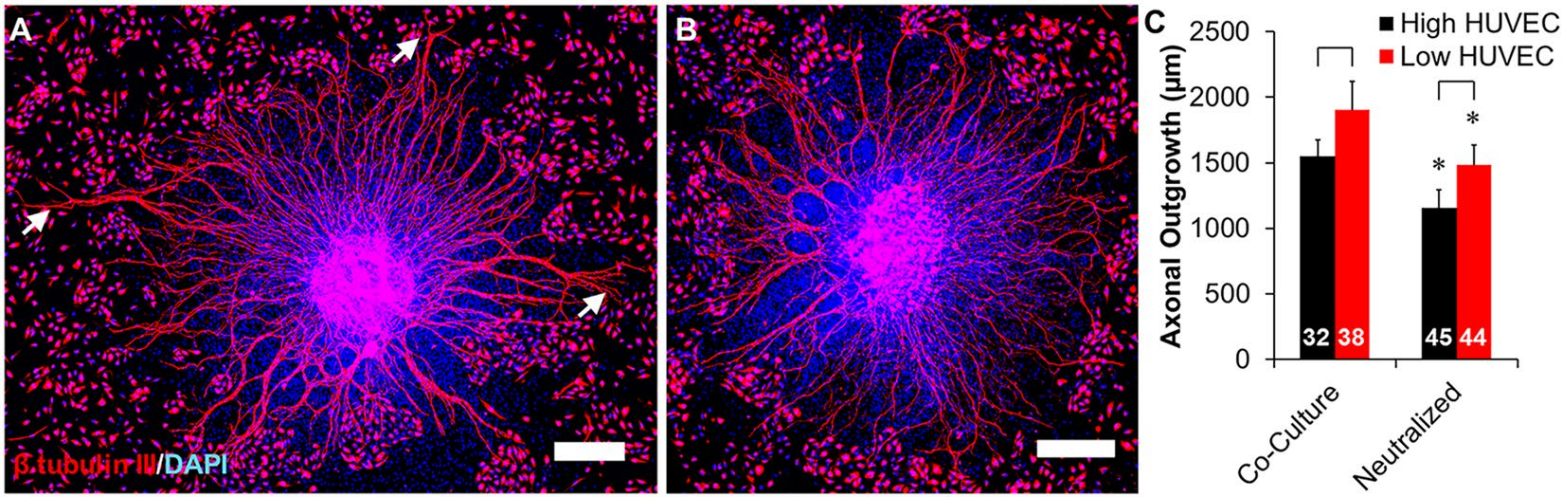
Neutralization of BDNF reduces DRG axonal growth in direct co-culture with HUVECs. DRG explants were cultured on (**A**) HUVECs and (**B**) HUVEC cultures supplemented with 125 ng/mL of BDNF neutralizing antibody. DRG-HUVEC co-cultures without neutralizing antibody displayed asymmetrical growth patterns, with long axons growing towards clusters of HUVECs (arrows). Supplementation of cultures with neutralizing antibodies restored symmetrical growth patterns. Scale = 200 µm. (**C**) Quantification of DRG axonal outgrowth from explants cultured with high or low density of HUVECs and with or without BDNF neutralizing antibodies. Neutralization of BDNF significantly reduced axonal outgrowth, while low density seeding conditions supported more axonal outgrowth independent of the addition of the neutralizing antibody. Data are presented as mean ± standard error. Brackets (p < 0.001) indicate significance between HUVEC densities and ***(p < 0.001) indicate significance between cultures with and without neutralization antibodies as determined by two-tailed Student’s t-tests (DRG sample size for each group shown in bars from 4 independent replicates).

Axons extending from DRG bodies grown in co-culture with HUVECs exhibited typical asymmetric growth towards clusters of HUVECs (Fig. 6A, arrows), and DRGs grown in co-culture with BDNF neutralizing antibodies showed radially symmetric growth rather than preferential growth towards any cell clusters (Fig. 6B). As shown in Fig. 1D, axonal growth was significantly longer when DRGs were cultured on low density HUVECs compared to DRGs cultured on high density HUVECs (Fig. 6C). Neutralizing antibodies significantly decreased axonal growth by 25% for co-cultures with both high and low density of HUVECs. Interestingly, axonal outgrowth remained significantly longer on low density HUVECs than cultures on high density HUVECs during treatment with neutralizing antibodies. To ensure that the concentration of neutralizing antibody completely knocked down BDNF activity, DRGs were cultured in conditioned medium or in co-culture with two times the concentration of neutralizing antibody. There were no differences in axonal outgrowth between neutralized conditions with conditioned medium or in co-culture with HUVECs (Supplemental Figure S2).

### Low-Density HUVECs Secrete Additional Neurotrophic Factors

**Figure 7:**
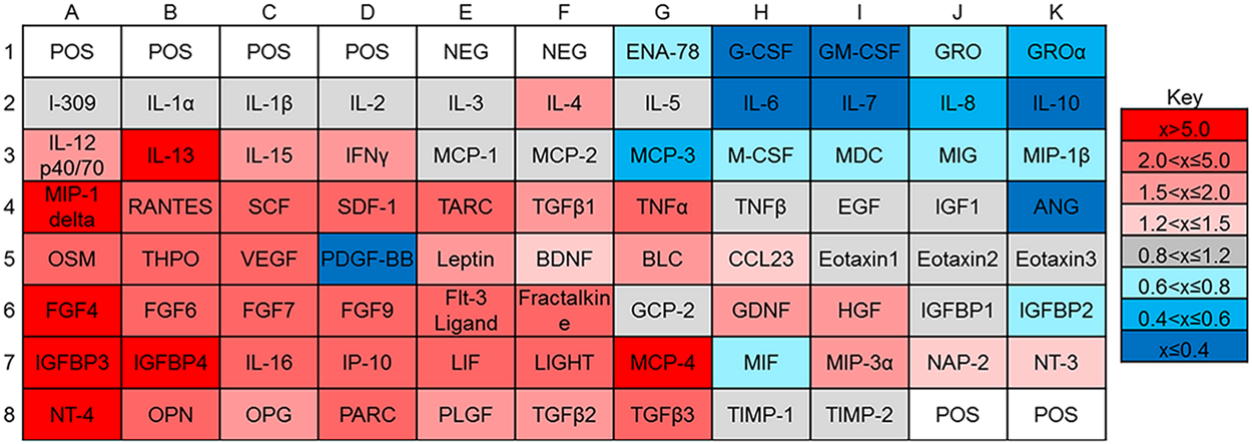
Protein expression is dependent on the concentration of HUVECs in culture. Protein expression of low density HUVEC monocultures (1,000 cell/cm^2^) were normalized against high density HUVEC monocultures (10,000 cell/cm^2^). In all cases, red indicates overexpression of indicated proteins and blue indicates underexpression of indicated proteins, with gradations of each color indicating relative amount of over/under expression. POS and NEG wells are internal controls within the cytokine array assay.

To determine how low density HUVECs enhanced axonal outgrowth with respect to high density HUVECs, a commercially available cytokine array was used to determine any differences in the secretome of these cell populations. There were a large number of angiogenic factors that were overexpressed, including SDF1, VEGF, FGF4, and FGF6 (Fig. 7). Interestingly, there were also several neurotrophic factors that were overexpressed in low density HUVEC cultures, including BDNF, GDNF, and NT4.

### HUVECs Enhance Axonal Growth from Mammalian DRGs

**Figure 8:**
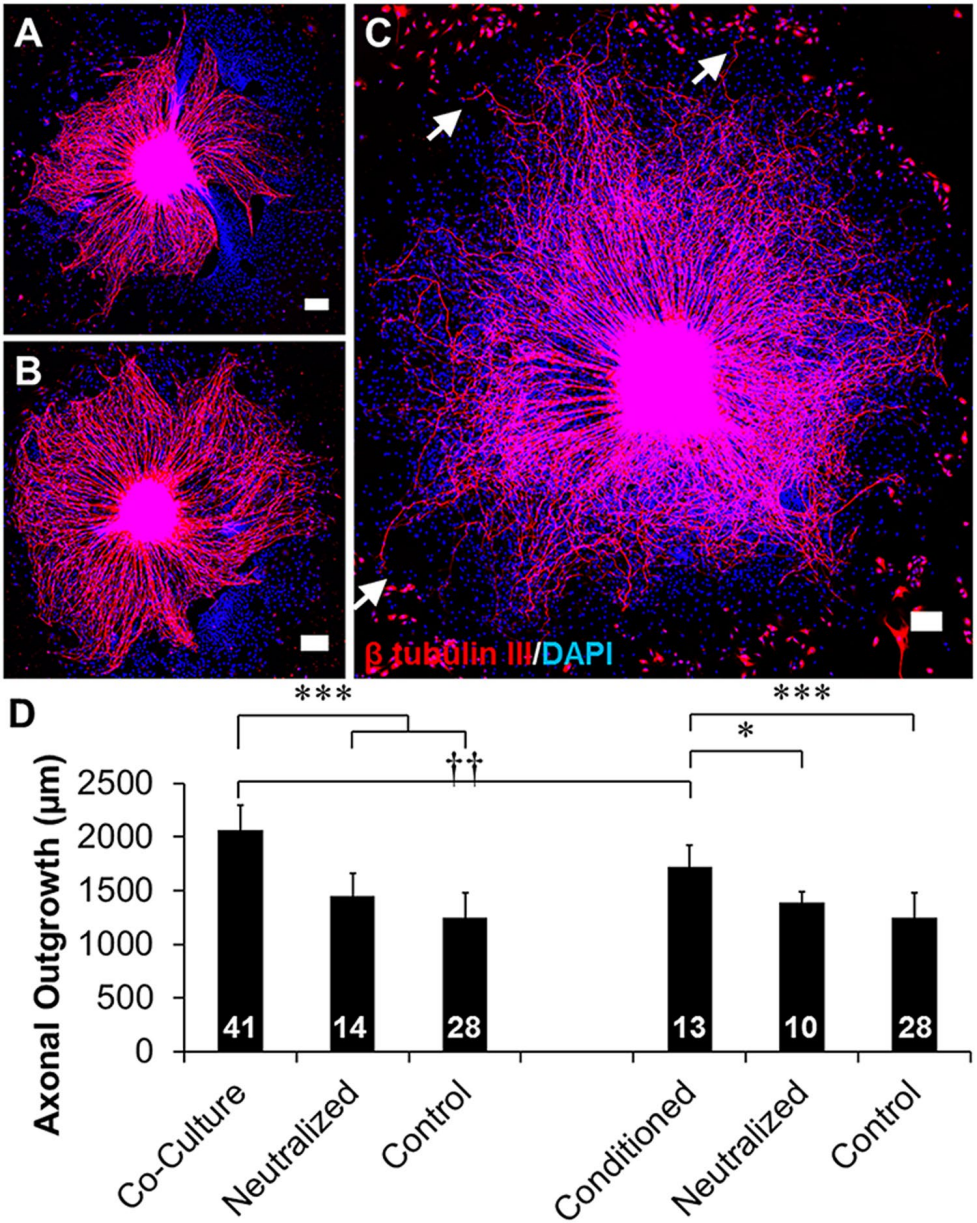
HUVEC co-culture and HUVEC-conditioned medium significantly enhanced rat DRG (rDRG) axonal growth. To confirm that HUVEC mediated axonal growth was applicable to mammalian systems, rDRG explants were cultured (**A**) on PDL wells (control), (**B**) in HUVEC-conditioned medium (conditioned), and (**C**) in direct co-culture with HUVECs (co-culture). Axons grew towards clusters of HUVECs when cultured in direct contact with HUVECs (arrows), and axons from DRGs grown on PDL well or with HUVEC-conditioned medium displayed radially symmetrical growth. Scale = 200 µm. Axonal outgrowth from (**D**) direct co-culture between low densities of HUVECs and rDRGs and from HUVEC-conditioned medium is significantly higher than control axons cultured on PDL wells (controls) and cultures supplemented with neutralizing antibody. Additionally, direct co-culture of HUVECs and rDRGs supported significantly longer axonal outgrowth than outgrowth from rDRGs cultured in HUVEC-conditioned medium. Data are presented as mean ± standard error. *(p < 0.05), ***(p < 0.001) and brackets indicate significance from indicated treatments by one-way ANOVA with Holm-Sidak post hoc analysis and ^††^(p < 0.01) indicates significance between indicated treatments as determined two-tailed Student’s t-test (DRG sample size for each group shown in bars from 2–3 independent replicates).

To confirm that the findings that human endothelial cells can support axonal growth in mammalian systems as well as avian systems, rat DRGs (rDRGs) were grown in direct co-culture with HUVECs or with HUVEC-conditioned medium. Axons extending from rDRGs cultured on PDL grew in radial symmetry from the rDRG body (Fig. 8A, control). rDRGs cultured in HUVEC-conditioned medium displayed enhanced axonal growth with an increased number of axonal projections while maintaining radial symmetry (Fig. 8B). Asymmetric growth of rDRGs was observed when co-cultured with HUVECs, with axons extending towards HUVECs (Fig. 8C). As with rDRGs cultured in HUVEC-conditioned medium, rDRG-HUVEC co-cultures displayed an increased number of longer axonal projections when compared to control rDRGs. Axons extending from rDRGs cultured in co-culture or with conditioned medium from HUVECs exhibited increased branching with respect to control rDRGs. Axons extending from rDRGs were significantly longer when cultured in HUVEC co-cultures or HUVEC-conditioned medium with respect to controls (Fig. 8D). Additionally, axons were significantly longer when cultured directly in co-culture with HUVECs rather than indirectly with HUVEC-conditioned medium. Supplementation of rDRG cultures with BDNF neutralizing antibody eliminated the enhanced outgrowth observed in direct co-culture with HUVECs as well as in culture with HUVEC-conditioned medium. Axonal outgrowth from rDRGs in neutralized cultures was approximately the same as outgrowth observed from controls.

## Discussion

The goal of this study was to investigate the ability of HUVECs to enhance the axonal growth of DRGs. Neural and endothelial structures develop along similar locations during embryonic development, and it was hypothesized in this study that endothelial cells secrete factors that would stimulate axonal growth. HUVECs were found to secrete factors that have been shown to be involved in peripheral nerve growth and maintenance in physiologically relevant concentrations capable of stimulating DRG growth. Specifically, we identified that HUVECs secreted comparable amounts of BDNF as reported in the literature^24, 25^, and that this factor is necessary for HUVEC-mediated increases in axonal growth. DRGs were co-cultured on myoblasts to determine if other cell types originating from a mesodermal lineage could enhance axonal growth. Our results confirmed that specifically endothelial cells, as opposed to cells of a mesodermal lineage, were capable of increasing axonal growth.

While co-culturing DRGs with myoblasts did not enhance axonal growth, immunohistochemical results displayed the formation of neuromuscular junction (NMJ) structures between these cell types. DRGs have previously been shown to form NMJ-like structures with myoblasts via scanning electron microscopy^26^, although the presence of biological markers was not investigated. In this study, we identified the presence of nicotinic acetylcholine receptors along the neurons as well as the body of the myoblasts. Importantly, we observed punctate staining that co-localized to both the axons and the myoblasts. These data suggest that DRGs can develop intercellular junctions such as synapses, both through identifying nicotinic acetylcholine receptors as well as identifying NMJ-like structures using microscopy in as little as 4 days of co-culture. The formation of functional NMJ structures in *in vitro* models of skeletal muscle could greatly enhance the understanding of how these structures form, affect myotube formation, and influence skeletal muscle tissue regeneration.

Normalization of the factors detected in the cytokine array from the DRG-HUVEC co-culture with the HUVEC monoculture conditions revealed several factors that may have been produced (overexpressed) or consumed (underexpressed) by the DRGs. There was a significantly higher expression of SDF1, FGF6, IL10, and IL4 in the co-culture condition from the neural population. While it is our hypothesis that these factors were secreted by the explants, we cannot exclude the possibility that the HUVECs have changed their secretion profile in the presence of DRG explants. SDF1 is a potent angiogenic factor that has been shown to enhance angiogenesis *in vivo* despite its limited lifespan in the bloodstream^27, 28^. FGF6 is thought to be localized to skeletal muscle^29^, and has been demonstrated to be important in myoblast proliferation and in skeletal muscle regeneration overall^30, 31^. IL10 and IL4 are cytokines that have been shown to drive macrophages towards the pro-regenerative M2 phenotype^32–34^. Taken together, the overexpression of these factors suggests that neural populations may indeed play an active role in local tissue remodeling *in vivo*. The ability of neural populations to stimulate angiogenesis *in vivo* could be a powerful way to direct vascularization of tissues experiencing peripheral nerve damage. Co-signaling between neural and endothelial populations could ultimately result in the re-innervation and vascularization of damaged tissues, such as skeletal muscle, through the active and direct recruitment of one of these cell types, thus simplifying design strategies for tissue engineering. Further, evidence that regeneration of the peripheral nervous system, as shown in our work with DRG axonal outgrowth, may modulate the inflammatory response of macrophages towards an M2 phenotype suggests that strategies to direct re-innervation can also direct the immune response towards a pro-regenerative state.

Significantly underexpressed cytokines and growth factors from the neural population include BDNF, FGF4, IL7, MIF, OPN, and PDGF-ββ. Because they were underexpressed in comparison to HUVEC monocultures, we hypothesized that these factors were consumed by the neural population. Of these factors, only BDNF significantly enhanced axonal growth. BDNF is a neurotrophic factor that has been shown to support neural cell growth and survival, and has been shown to be essential for endogenous peripheral nerve regeneration^9^. It is interesting to note that the remainder of these factors did not significantly contribute to axonal growth independently, despite previous work suggesting that IL7, predominately involved in inflammatory signaling, has been shown to increase survival in neurons from the central nervous system, resulting in an increase in axonal growth^35^. MIF has also been shown to regulate pain sensation and enhance axonal growth in DRG neurons, despite not affecting axonal growth in these experiments^36^. Osteopontin has a variety of roles in tissue development including inflammation in tissue regeneration and stimulating neural growth in neurons^37, 38^. However, it seems that the ability of OPN to stimulate axonal growth is limited to neurons in the central nervous system and may act more directly to stimulate the survival of these cells rather than directly encourage axonal growth^39^. PDGF-ββ has been shown to enhance the survival of Schwann cells, which are present in DRG explants^40^. Schwann cells are essential for the maintenance of peripheral nerves, both for their function and regeneration. One reason that PDGF-ββ may not have affected axonal growth in DRG explants could be that the culture environment was already conducive to Schwann cell survival. Another possible explanation could be that these other growth factors work in combination with BDNF, or each other, to enhance axonal growth. Supplementation with IL7 resulted in significantly longer axons when grown in EGM-2 medium rather than DMEM, suggesting that IL7 may be synergistically signaling DRG explant tissue with factors present specifically in EGM-2 medium, such as VEGF or epidermal growth factor (EGF). BDNF neutralization studies did not result in a complete knockdown of axonal growth when added to direct co-cultures, which suggests that some of these other growth factors released by HUVECs might synergistically assist in axonal growth, either by stimulating the neurons directly or indirectly through the supporting cells present in the explants.

It is interesting to note that a lower initial seeding density of HUVECs increased the axonal growth of neurons with respect to higher seeding densities. While there was less BDNF present in the medium of low density HUVECs with respect to high density HUVECs, there was more BDNF secreted per cell. This would suggest that the gradient, in addition to the concentration of factors, influenced axonal outgrowth. This phenomenon has been observed by studying the growth cone of axons, where direct application of factors to the growth cone will affect neuron attraction or repulsion to this directed stimulus^41, 42^. Additionally, we observed significantly longer axonal outgrowth from low density HUVECs with respect to high density HUVECs grown in co-culture with DRGs with and without treatment with BDNF neutralizing antibodies. Taken together, these data suggest that low density HUVECs may be secreting additional factors that stimulate axonal outgrowth. To investigate this hypothesis, we analyzed the secretome of low density and high density HUVEC cultures and found that low density HUVECs secreted more angiogenic and neurotrophic factors. The overexpressed factors included GDNF, a potent factor used in a variety of *in vivo* studies to enhance peripheral nerve growth^5, 43^, and NT4, another neurotrophic factor implicated in neural survival and growth^7^. These factors likely are acting independently to BDNF, as neutralization of BDNF in co-culture maintained the significant differences observed in axonal outgrowth. An alternate hypothesis could be that low density HUVECs are also depositing extracellular matrix that stimulates axonal growth. Regardless, gradient based guidance of neurons towards endothelial cells would be an essential concept to consider that blood vessels might be serving as a template for axonal migration throughout the periphery of mammals through development. In this way, patterning or directing angiogenesis in the presence of certain cues, such as BDNF, could assist in peripheral nerve regeneration, especially in traumatic wounds such as volumetric loss injuries, where there is no regenerative template present.

Rat DRG explants behaved similarly to chick DRGs when grown on monolayers of HUVECs, confirming that stimulants for axonal growth in chick and rat DRGs are similar. HUVEC-mediated increases in axonal growth were completely attenuated when incubated with BDNF neutralizing antibodies, demonstrating that mammalian DRGs are also sensitive to HUVEC secreted BDNF. Surprisingly, there was a significant difference in the axonal growth between direct co-culture and indirect culture in HUVEC-conditioned medium. These findings further suggest that additional factors must be present in the direct co-culture condition that may be further enhancing HUVEC-secreted soluble factor-mediated axonal outgrowth. Likely this could be the secretion, and organization, of some matrix protein. The additional guidance of neurons to endothelial cells via matrix protein synthesis could also explain the close patterning of endothelial cells and neurons in capillary networks, and future work will be focused on identifying proteins that may be facilitating this directed guidance. These findings can inform future studies by patterning or directing angiogenesis *in vivo* to direct re-innervation in traumatic injuries or by strategically binding BDNF to biomaterials to direct peripheral nerve growth, and could also be used to develop novel model systems to study angiogenesis and neurogenesis *in vitro* to enhance current material-based interventions to regenerate large scale, traumatic injuries.

## Methods

### Cell Culture

Human umbilical vein endothelial cells (HUVECs, Lonza, Walkersville, MD) were cultured in complete EGM-2 medium (Lonza) according to the manufacturer’s instructions. Cells were incubated at 37 °C with 5% CO_2_ and maintained using standard cell culture techniques. Routine cell passage was conducted at 80–90% confluence using 0.25% trypsin-EDTA (CellGro). Cells were not used for experiments once they reached passage 11, and were typically between passages 6–10.

Immortalized mouse myoblasts (C2C12, ATCC, Manassas, VA) were cultured in complete Dulbecco’s modified Eagle Medium growth medium (DMEM; 1:1 (*v/v*) ratio of high glucose DMEM (Gibco BRL, Gaithersburg, MD) and Ham’s F12 (Gibco) supplemented with 4 mM L-glutamine and Ham’s F12 (Gibco) and 10% fetal bovine serum (FBS, Sigma, St. Louis, MO)). Cells were incubated at 37 °C with 5% CO_2_ and maintained at a density below 70% confluence using standard cell culture techniques. Routine cell passage was conducted using 0.25% trypsin-EDTA (CellGro, Manassas, VA). Differentiation was induced by culturing confluent C2C12s in differentiation medium (DM; 1:1 (*v/v*) ratio of high glucose DMEM and Ham’s F12 with 4 mM L-glutamine and 2% denatured horse serum (HyClone, Logan, UT)).

For co-culture studies, HUVECs were seeded into 24 wells at a high density (10,000 cell/cm^2^) or at a low density (1,000 cell/cm^2^) and cultured for three days prior to addition of dorsal root ganglia (DRGs). Myoblasts were seeded into 24 wells at a density of 5,000 cell/cm^2^ and cultured for three days in DMEM medium prior to being cultured in DM.

### DRG Isolation and Culture

All animal protocols were approved by the Institutional Animal Care and Use Committee (IACUC) at Tufts University, and all procedures and methods were performed in accordance with relevant institutional guidelines and regulations. Chicken DRGs were isolated from E8 chicken embryos (University of Connecticut, Poultry Farm, CT), as previously described^44^. Briefly, under aseptic conditions, tissue from the embryo was dissected away to expose the spinal column. DRGs were removed from each embryo using fine-pointed forceps and surrounding fascia were removed using forceps and scalpel blades. DRGs were cut in half using scalpel blades and cultured in DMEM or EGM-2 medium. Three DRGs were added to 24 wells that were pre-seeded with HUVECs or myoblasts, or were previously coated with poly-D-lysine (PDL, Sigma). Wells were incubated with 100 µg/mL PDL diluted in de-ionized (DI) water overnight at 4 °C and rinsed with phosphate buffered saline (PBS) to coat with PDL. DRG co-cultures were cultured for 4 days and then fixed with 4% paraformaldehyde (Boston BioProducts, Ashland, MA) for immunostaining. For conditioned medium studies, DRGs were seeded onto PDL well plates with medium conditioned by high density HUVECs. Medium was changed every other day to ensure that factors secreted by the HUVECs were present throughout the culture period of 4 days.

In some studies, neonatal rat DRGs (rDRGs) were seeded on PDL coated wells or HUVECs. E18 rat pups were euthanized via CO_2_ inhalation and decapitation, and rDRGs were isolated using similar dissection techniques as described for chick DRGs. As with chick DRGs, three rDRGs were added to 24 wells pre-seeded with HUVECs or pre-coated with PDL, cultured for 4 days and fixed with 4% paraformaldehyde.

### Cytokine Array for HUVEC Cytokine Secretion

To determine what cytokines were secreted by HUVECs and HUVEC-DRG co-cultures, a cytokine array was performed at the end of monocultures of HUVECs and co-cultures of HUVEC-DRGs. The supernatant of each condition was analyzed for 80 cytokines (Cat No. AAH-CYT-5; RayBiotech, Norcross, GA) according to the manufacturer’s instructions. Data were normalized first to internal controls in each array and then to arrays performed on blank EGM-2 medium incubated in parallel to the mono- and co-cultures. Finally, to determine what factors were secreted and consumed by DRGs in these co-culture conditions, data obtained from HUVEC-DRG co-culture experiments were normalized to monocultures of HUVECs.

### Growth Factor Supplementation Studies

To determine the effects of individual growth factors on axonal outgrowth, DRG explants were cultured on PDL coated wells in either DMEM or EGM-2 supplemented with growth factors. Mature brain-derived neurotrophic factor (BDNF), glial cell-derived neurotrophic factor (GDNF), vascular endothelial growth factor (VEGF), interleukin 7 (IL7), osteopontin (OPN), platelet derived growth factor ββ (PDGF-ββ), fibroblast growth factor 4 (FGF4), and macrophage migration inhibitory factor (MIF) were all purchased from Peprotech (Rocky Hill, NJ) and added to EGM-2 or DMEM at a concentration of 0, 5, 50, or 100 µg/mL. Two to three chick DRGs or rDRGs were added to each well, with each condition being run in duplicate. DRGs were cultured for 4 days without additional growth factor supplementation and subsequently fixed with 4% paraformaldehyde for immunocytochemical analysis.

### BDNF ELISA

To determine the amount of BDNF secreted by HUVECs, ELISAs were performed on HUVEC-conditioned medium at the end of monocultures cultured in parallel with co-culture experiments. An ELISA against BDNF (Abcam, Cambridge, MA) was performed according to manufacturer’s instructions with experimental conditions being performed in quadruplicate from at least two independent replicates.

### BDNF Neutralization Studies

To determine if BDNF was necessary for HUVEC-mediated axonal outgrowth, DRG-HUVEC co-cultures and DRGs cultured in HUVEC-conditioned medium were cultured in the presence of neutralizing antibody. BDNF neutralization antibody (Cat No. 500-P84; Peprotech) was added to cultures immediately before DRGs were seeded onto HUVECs (direct co-culture) or into HUVEC-conditioned medium every other day at a concentration of 125 ng/mL. At least 2 DRGs were seeded into each well and samples were run in quadruplicate for each replicate. Cultures were fixed after 4 days with 4% paraformaldehyde for immunocytochemical analysis.

### Immunocytochemistry

After four days of culture, DRGs and DRGs co-cultured with HUVECs were fixed in 4% paraformaldehyde and permeabilized with 0.1% Triton X-100 (Sigma), blocked using 5% bovine serum albumin (BSA), and immunostained with a primary antibody against β-tubulin III (1:500, Cat No. T2200; Sigma) and an Alexafluor 594 secondary (1:500, A-11072; ThermoFisher Scientific, Waltham, MA). DRG-myoblast co-cultures were immunostained with a primary antibody against myosin (1:50, MF20; Developmental Studies Hybridoma Bank, Iowa City, IA) and β tubulin III (1:500) as well as appropriate species-matched Alexafluor 488 and 594 secondaries (1:500, A-21202 and A-11072 respectively; ThermoFisher Scientific) and Alexafluor 647 tagged α-bungarotoxin to visualize neuromuscular junctions (1:250, B-35450; ThermoFisher Scientific). All cultures were counterstained with 4′,6-Diamidino-2-Phenylindole, Dilactate (DAPI; 1:2000, ThermoFisher Scientific).

### DRG Axon Length Measurements

Fluorescent images were obtained using a Keyence BZ-X700 microscope and associated software (Keyence, Elmwood Park, NJ). Multiple images were merged together to assemble composite images of each DRG explant. Merged images were opened in Image J (NIH) and the longest axon was recorded from its growth cone to the DRG body. In all cases, the observer was blinded to the treatment group that they were imaging and analyzing. The size of each DRG body was also recorded. Any DRG that did not have an easily defined DRG body, or a body that was smaller than 150 µm was omitted from further analysis.

### Statistical Analyses

Statistical analyses were performed using a one-way analysis of variance (ANOVA) with p < 0.05 indicating significant differences between groups using SigmaPlot 13.0 software (Systat Software, Inc., San Jose, CA). For post hoc analyses, a Holm-Sidak pairwise multiple comparison test was performed to determine significance between experimental groups using an overall significance level of p < 0.05. Where indicated, a Student’s t-test was performed where differences between conditions were considered significant at p < 0.05. Data are reported as means ± standard errors.

### Data Availability

The authors declare that the data supporting the findings of this study are either available within the article or from the corresponding author upon request.

## Electronic supplementary material

Grasman_Supplemental_Info
