# Supplementary material for: Human endothelial cells secrete neurotropic factors to direct axonal growth of peripheral nerves
Source: Sci Rep. 2017 Jun 22;7:4092. doi: 10.1038/s41598-017-04460-8 (PMC5481420; doi:10.1038/s41598-017-04460-8)
Supplement: Supplementary file 1 — Grasman_Supplemental_Info [file 41598_2017_4460_MOESM1_ESM.pdf]

SUPPLEMENTAL INFORMATION

Human endothelial cells secrete neurotropic factors to direct axonal growth of peripheral nerves

Jonathan M. Grasman and David L. Kaplan*

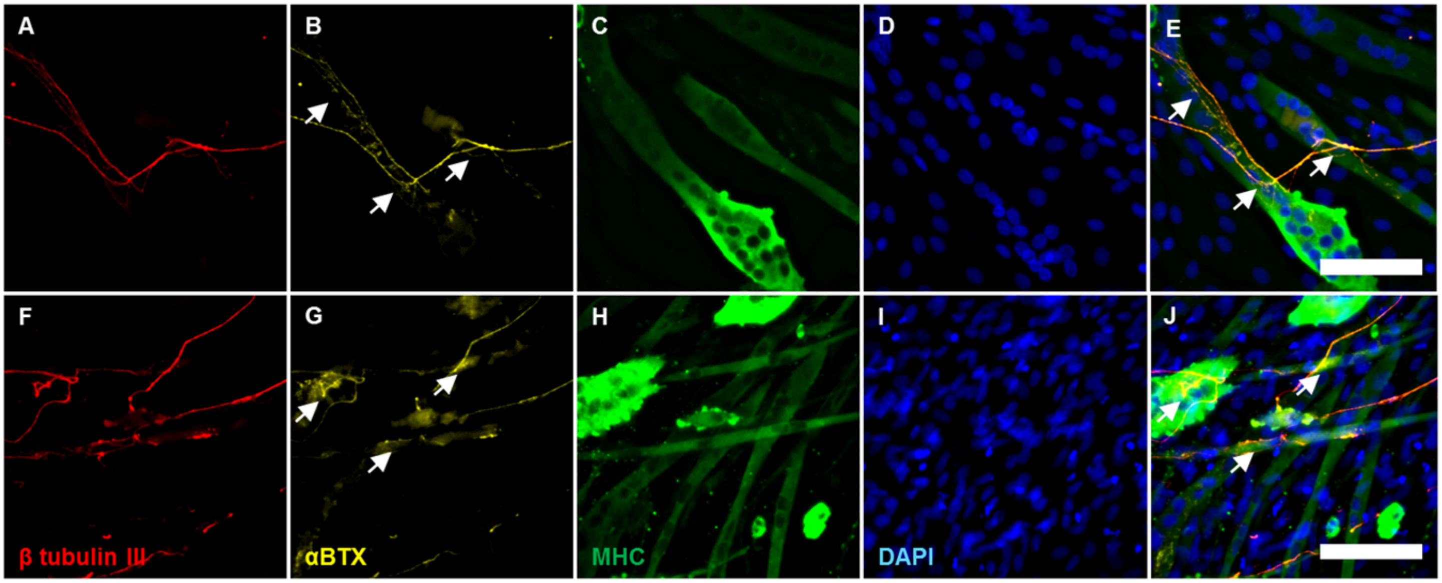

Supplemental Figure S1. Co-culture of myoblasts and chick DRGs developed neuromuscular junction structures. DRGs were seeded onto confluent myoblasts in differentiation medium and after 4 days co-localized α-bungarotoxin (αBTX), a marker for nicotinic acetylcholine receptors, along both neurons and myotubes. Arrows indicate regions of staining on myoblasts that appear to be neuromuscular junctions. Scale = 50 µm.

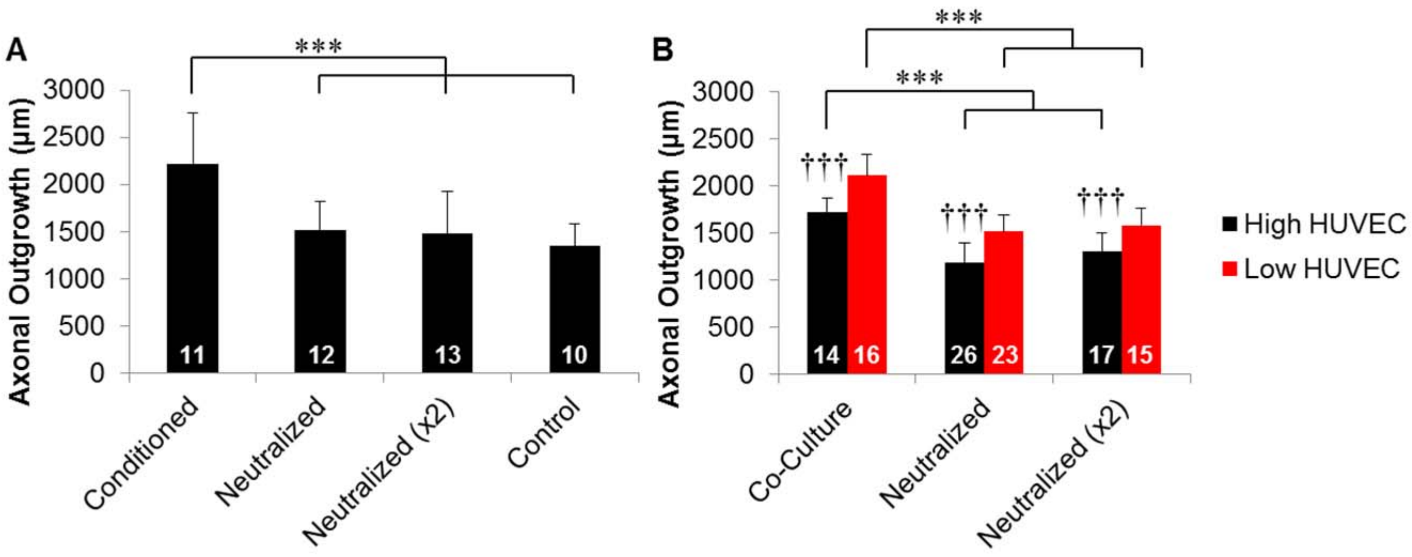

Supplemental Figure S2. Higher concentrations of BDNF neutralizing antibody do not affect axonal outgrowth. DRGs grown in (A) HUVEC-conditioned medium and (B) direct co-culture were cultured with neutralization antibody as described in the manuscript (125 ng/mL) or with twice the concentration of antibody (250 ng/mL). Enhanced neutralization did not lower axonal outgrowth with respect to normal neutralized conditions or the control (DRGs grown in monoculture). Data are presented as mean ± standard error. *** (p<0.001) and brackets indicate significance from other treatments as determined by one-way ANOVA with Holm-Sidak post hoc analysis and ††† (p<0.001) indicates significance with low density HUVECs as determined by two-tailed Student's t-test (DRG sample size for each group shown in bars from 2 independent replicates).
